# Prenatal stress and gestational epigenetic age: No evidence of associations based on a large prospective multi-cohort study

**DOI:** 10.21203/rs.3.rs-4257223/v1

**Published:** 2024-07-04

**Authors:** Chris Murgatroyd, Kristina Salontaji, Dinka Smajlagic, Christian Page, Faye Sanders, Astanand Jugessur, Robert Lyle, Stella Tsotsi, Kristine Haftorn, Janine Felix, Esther Walton, Henning Tiemeier, Charlotte Cecil, Mona Bekkhus

**Affiliations:** Manchester Met University; Norwegian Sequencing Centre; Norwegian Sequencing Centre; Norwegian Sequencing Centre; Erasmus MC; Harvard T. Chan School of Public Health; Erasmus Medical Centre, University Medical Center Rotterdam

**Keywords:** Epigenetic clock, prenatal stress, ALSPAC, MoBa, Generation R

## Abstract

Psychological stress during pregnancy is known to have a range of long-lasting negative consequences on the development and health of offspring. Here, we tested whether a measure of prenatal early-life stress was associated with a biomarker of physiological development at birth, namely epigenetic gestational age, using foetal cord-blood DNA-methylation data.

Longitudinal cohorts from the Netherlands (Generation R Study [Generation R], n = 1,396), the UK (British Avon Longitudinal Study of Parents and Children [ALSPAC], n = 642), and Norway (Mother, Father and Child Cohort Study [MoBa], n1 = 1,212 and n2 = 678) provided data on prenatal maternal stress and genome-wide DNA methylation from cord blood and were meta-analysed (pooled n = 3,928). Measures of epigenetic age acceleration were calculated using three different gestational epigenetic clocks: “Bohlin”, “EPIC overlap” and “Knight”.

Prenatal stress exposure, examined as an overall cumulative score, was not significantly associated with epigenetically-estimated gestational age acceleration or deceleration in any of the clocks, based on the results of the pooled meta-analysis or those of the individual cohorts. No significant associations were identified with specific domains of prenatal stress exposure, including negative life events, contextual (socio-economic) stressors, parental risks (e.g., maternal psychopathology) and interpersonal risks (e.g., family conflict). Further, no significant associations were identified when analyses were stratified by sex. Overall, we find little support that prenatal psychosocial stress is associated with variation in epigenetic age at birth within the general paediatric population.

## Introduction

Prenatal exposure to maternal stress is associated with increased risk for child emotional and behavioural symptoms as well as poor mental (e.g., anxiety and depression [1]) and physical (e.g., cardiovascular and metabolic disease [2–4]) health outcomes in adulthood. One proposed pathway through which exposure to stressors and resultant stress hormones could increase risk for this wide range of health problems is the foetal programming of adult disease [5]. First characterized by Barker [6], it is thought to occur through exposure of the foetus to a less than-optimal in-utero environment that may alter the timing and rate of growth and development, resulting in accelerated biological aging; a concept based on life history theory [7].

Evidence for accelerated biological aging following prenatal stress has been found across a variety of metrics. For example, prenatal stress is associated with earlier pubertal timing [8] and accelerated cellular aging reflected by shorter telomere length [9]. Neonatal neuroimaging studies support a specific contribution of prenatal maternal distress to infant neurodevelopment [10], with studies finding impacts on amygdala-PFC functional connectivity [11] and cortical thinning across development [12].

More recently, gestational epigenetic clocks have been developed that measure patterns of DNA methylation (DNAm) in neonatal tissues to compute measures of gestational age. Calculating the difference between epigenetically-estimated gestational age and clinically-estimated gestational age can give a measure of gestational epigenetic age acceleration or deceleration. This can serve as a surrogate indicator of the pace of physiological development of a neonate, with higher epigenetic age acceleration values potentially indicating more developmental maturity in neonates [13]. For example, epigenetic age acceleration or deceleration in neonates has been associated with numerous prenatal factors such as maternal pre-pregnancy obesity and cardiometabolic factors [14] maternal smoking [15] and maternal age [16], as well as infant outcomes, such as birth weight, birth length, and head circumference [17].

Numerous studies have found associations between psychosocial stress and accelerated epigenetic aging across the lifespan (for a review, see [18]). For example, an accelerated epigenetic age has been found in blood from adults with an increased lifetime stress exposure [19] and in mothers exposed to stress a year before pregnancy [20] or mothers who experienced trauma [21]. Studies have also shown associations between accelerated epigenetic agen and stress during childhood and postnatal early life, while exposure to adverse childhood experiences were reported to be associated with accelerated epigenetic age in middle-aged adults [22]. Such accelerated epigenetic aging has been further linked to negative mental and physical health outcomes [23]. For example, one study found that children diagnosed with an internalizing disorder who also had experienced maltreatment showed an accelerated epigenetic age compared to children with no internalizing disorder [24]; however the the association was moderated by the degree of malnutrition.

Together, these studies consistently point to an association between *postnatal* stress exposure and accelerated epigenetic age at various developmental and life stages. However, the picture is less clear when investigating the effects of *prenatal* stress exposure on epigenetic age, with both negative and positive associations reported in the literature. For example, Suarez et al. reported a significant association between prenatal maternal depression and lower gestational epigenetic age acceleration at birth, which was in turn associated with developmental problems in boys [25]. Similarly, studies by Koen et al. [26] in 271 newborns and Katrilini et al. [20] in 89 new-borns both found lower gestational epigenetic age in those from mothers who displayed symptoms of PTSD during pregnancy, suggesting possible delayed foetal development [20]. In contrast, a study in Brazil in 83 newborns found an association between gestational epigenetic age acceleration and maternal negative feelings related to pregnancy, though this was not associated with any other psychosocial variables. They further found that gestational epigenetic age acceleration could be predicted by epigenetic scores of low-grade inflammation and glucocorticoid exposure [27]. A study in the Democratic Republic of Congo, on 141 newborns, also found that those prenatally exposed to general trauma and war trauma displayed epigenetic age acceleration at birth. However, they did not use gestational age-specific clocks [21]. Finally, prenatal maternal anxiety was found to predict accelerated epigenetic aging across infancy and mid-childhood in two independent cohorts with increased sensitivity in males [28].

In summary, several studies have reported a link between prenatal stress exposure and epigenetic age at birth, supporting its potential role as a biomarker and mechanism linking stress exposure with poor physiological, neurodevelopmental and health outcomes later in life. However, the direction of associations and the implications for health outcomes have been largely inconsistent. This could be due to a number of factors, including differences in the type of exposure examined (e.g., maternal depression, anxiety, trauma), epigenetic clock used, and sample characteristics (e.g., community-based vs high-risk samples). Furthermore, studies have been mainly based on single datasets of modest sample size, limiting statistical power and the ability to identify robust, generalizable findings. Overall, little is known about how different types of prenatal stress exposures cumulatively and independently associate with differences in offspring gestational epigenetic age at birth in the general population, and whether associations differ by offspring sex.

To address these questions, we pooled data from 3928 mother-offspring dyads from three independent European prospective population-based birth cohorts. We created comparable composite scores of prenatal stress across the cohorts, comprised of multiple stress domains (negative life events, contextual risks, parental risks, and interpersonal risks), which enabled us to examine both the cumulative and independent effects of different prenatal stressors on epigenetic gestational age. To maximize comparability with the existing literature and enable a comprehensive evaluation of stress- epigenetic age associations, we calculated epigenetic age estimates using three different clocks that have been developed for use with neonatal cord blood, namely “Knight” [29], “Bohlin” [30] and “EPIC overlap” [31]. For each of these, we measured both epigenetic estimates of gestational age, as well as gestational age deceleration or acceleration (i.e., the difference between chronological and epigenetic age). Cohort-specific results were meta-analysed to increase statistical power and identify robust associations. Finally, we repeated analyses stratified by offspring sex, to establish whether prenatal stress may be associated with epigenetic gestational age estimates differently for boys and girls.

## Methods

### Study cohorts

The analyses in this study were conducted using data from three prospective population-based cohorts: the Dutch Generation R Study (Generation R) [32], the British Avon Longitudinal Study of Parents and Children (ALSPAC) [33, 34], and the Norwegian Mother, Father, and Child Cohort Study (MoBa) [35]. In total, this study included pooled results from 3,928 children.

Generation R. Pregnant women residing in the study area of Rotterdam, the Netherlands, expected to deliver between April 2002 and January 2006, were invited to enrol in the Generation R Study [36]. The study was conducted in accordance with the World Medical Association Declaration of Helsinki and has been approved by the Medical Ethics Committee of Erasmus MC, University Medical Center Rotterdam (MEC 198.782/2001/31). Informed consent was obtained from all participants. The Generation R Study includes data from 9,749 live-born children. DNAm data at birth are available for 1,396 children who also had at least 50 per cent of items included in the prenatal stress measure.

MoBa. Participants include mothers and their children, residing in Norway with an expectation to deliver between 1999 and 2008; the participation rate among the mothers was 41% and from 2003, the fathers were also included [37, 38]. Ethical approval for the MoBa study was obtained from the Regional Committees for Medical and Health Research Ethics (REK- 2009/1899–7), and the current sub-study (REK: 2020/185800). The MoBa cohort includes approximately 114,500 children, 95,200 mothers and 75,200 fathers. The current study is based on version 12 of the quality-assured data files released for research in 2020 and comprises different subsamples with DNAm data. In our analysis, we only included subsamples that (i) comprised randomly selected participants and (ii) have not been previously used to develop the epigenetic clocks used in this study (i.e., the Bohlin clock has been trained on MoBa 1 data). Based on this selection, we included the MoBa 1 subsample with DNAm data from 1,212 children and MoBa 2 with data from 678 children (*see* Supplementary Methods).

ALSPAC. Pregnant women residing in the study area of the former county Avon in the United Kingdom with an expected delivery date between April 1991 and December 1992 were invited to enroll in ALSPAC [39]. Ethical approval for the ALSPAC study was obtained from the ALSPAC Ethics and Law Committee and the Local Research Ethics Committees. The ALSPAC study includes data from 14,541 live-born children. DNAm data at birth was available for 642 children who also had at least 50% of stress measures for the prenatal period. Study data were collected and managed using REDCap electronic data capture tools hosted at the University of Bristol, UK. REDCap (Research Electronic Data Capture) is a secure, web-based software platform designed to support data capture for research studies [40]. Please note that the study website contains details of all the available data through a fully searchable data dictionary and variable search tool” at the following webpage: http://www.bristol.ac.uk/alspac/researchers/our-data/

### Prenatal psychosocial stress assessment

A cumulative prenatal stress score was created based on our previous work [41]. The score consists of the following domains: (i) life events (e.g., death of a parent or pregnancy complications), (ii) contextual risk (e.g., financial difficulties or neighbourhood problems), (iii) parental risk (e.g., parental criminal record or parental psychopathology), and (iv) interpersonal risk (e.g., family conflicts or loss of a friend). Relevant items were dichotomized into ‘risk’ (1) or ‘no risk’ (0) and the mean averaged to form the scores for each domain. The cumulative score for prenatal stress was then computed by summing its respective domain scores. Both the cumulative score and its four individual stress domains have been harmonized across all three cohorts included in the present study. Further details of included items and the time points at which they were collected in Generation R and ALSPAC can be found here: https://github.com/SereDef/cumulative-ELS-score; for MoBa (https://www.fhi.no/en/ch/studies/moba/for-forskere-artikler/questionnaires-from-moba/), and a detailed description in see [42].

### DNA methylation

In all cohorts, cord blood was drawn at birth and 500 ng of DNA were bisulphite converted using the EZ-96 DNA Methylation kit (Zymo Research Corporation, Irvine, USA). The Generation R and ALSPAC samples were processed with the Illumina Infinium HumanMethylation450 BeadChip, whereas the MoBa samples were processed with either HumanMethylation450 BeadChip (MoBa 2) or Illumina MethylationEPIC 850K array (Illumina Inc., San Diego, CA). Detailed control steps and normalization procedures have been described previously [43–46]. In short, in Generation R, the CPACOR workflow was used for quality control and normalization [46]. Arrays with observed technical problems such as failed bisulphite conversion, hybridization, or extension as well as arrays with a sex mismatch were removed. Probes that had a detection p-value above background ≥ 1E-16 were set to missing per array. Arrays with a call rate > 95% per sample were included and quantile normalized (as described previously [43]). In ALSPAC, the *meffil* package [47] was used for quality control. Samples with mismatched genotypes, mismatched sex, incorrect relatedness, low concordance with samples collected at other time points, extreme dye bias, and poor probe detection were removed and carried forward into normalization. In MoBa, all duplicates were removed and arrays not fulfilling the 5 % detection p valu were excluded [48]. Within-array normalization of type I and II probes was performed using BMIQ from the R package watermelon [49]. Detailed pre-processing steps have been published previously [44, 45].

### Age estimates

Gestational age at birth was assessed from midwife or obstetric records. Gestational epigenetic clocks make use of a select number of CpG sites that are strongly correlated with gestational age at birth. The gestational epigenetic clock developed by Bohlin *et al*. [30] estimates epigenetic age based on DNAm levels at 96 CpG sites from the HumanMethylation450 BeadChip that were selected using Lasso regression and the predictions were tested in an independent sample. The epigenetic clock developed by Knight *et al*. [29] estimates epigenetic age at birth based on DNAm levels at 148 CpG sites from the HumanMethylation450 BeadChip and the HumanMethylation27 BeadChip that were selected using elastic net regression and the predictions tested in holdout samples. The EPIC overlap clock developed by Haftorn *et al*. [31] is based on CpGs that can be found both on HumanMethylation450 BeadChip array and on the Illumina MethylationEPIC 850K array. It estimates epigenetic gestational age based on 173 CpG sites that were selected using Lasso regression and the predictions tested in holdout samples. The *methylclock* package in R 4.1.1 was used to calculate gestational epigenetic age measures and to impute missing values if less than 20% were missing based on the Bohlin, Knight and EPIC overlap clocks. We adjusted the package to also calculate the EPIC overlap clock, as this clock had not yet been implemented in the *methylclock* package at the time of conducting this study. For each clock, we computed the epigenetically-estimated age and adjusted for clinically-estimated gestational age at birth in each model to optimally account for the correlation between (i) epigenetic gestational age estimates and gestational age and (ii) gestational age and other covariates included in each model.

### Covariates

Covariates included in the primary analysis were sex, gestational age at birth, cell-type proportions, and batch effects. Maternal prenatal smoking, maternal alcohol consumption, maternal pre-pregnancy BMI, delivery method, maternal age at delivery, and parity were included in an extended model. The birth weight of the child was included in a third model in addition to the extended model covariates as it may be part of the causal pathway. Missing covariates were imputed using the Multivariate Imputations by Chained Equations “*mice*” package in R. Self-administered questionnaires completed by the mothers during pregnancy were used to obtain information on maternal covariates and information on child sex, and birth weight was obtained from midwife and hospital records. Cell-type proportions were estimated using a cord blood-specific reference set in the “FlowSorted.CordBlood.Combined.450k” Bioconductor package [50]. This reference set includes CD8 + T cells, CD4 + T cells, natural killer cells, B cells, monocytes, granulocytes, and nucleated red blood cells.

### Statistical analyses

The association of prenatal stress exposure with epigenetic clock estimates at birth was tested within each cohort using robust linear regression models (*rlm* package) in R version 4.1.1. We computed the following models with epigenetic clock estimates as an outcome and (i) total prenatal stress score or (ii) the separate prenatal stress subdomains modelled together as exposures, to estimate their independent effect. We also computed the models mentioned above separately for boys and girls to investigate potential sex-specific associations. Analyses were run with a core set of covariates, and with an extended set of covariates, as described above. Missing data on covariates and the prenatal stress score were imputed using multiple imputations, with the *mice* package. Subdomains and the total ELS score were not imputed directly. Instead, separate items pertaining to each subdomain were imputed separately first, then subdomain scores and finally the total ELS score was calculated. Model parameters in each imputed dataset (out of 30 datasets) were fitted and then pooled according to Rubin’s rulesv[51]. Cohort-specific results for each epigenetic clock were meta-analysed using the rma.uni function of the *metafor* package using a fixed-effects model and inverse-variance weighting.

## Results

### Subject characteristics

Table 1 shows maternal and child characteristics based on non-imputed data for each cohort. There was no substantial difference in sample characteristics in terms of descriptive statistics between the imputed and non-imputed datasets.

### Associations between clinically-estimated and epigenetically-estimated gestational age

The correlation between clinical and epigenetic measures of gestational age was high across cohorts for the Bohlin clock (*r*_*GneR*_ = 0.70; *r*_*Moba*_ = 0.74; *r*_*MoBa2*_ = 0.72; and *r*_*ALSPAC*_ = 0.61), as well as for the EPIC overlap clock (*r*_*GenR*_ = 0.71; *r*_*Moba*_ = 0.74; *r*_*MoBa2*_ = 0.72; and *r*_*ALSPAC*_ = 0.58), whereas it was moderate for the Knight clock (*r*_*GenR*_ = 0.46; *r*_*Moba*_ = 0.57; *r*_*MoBa2*_ = 0.54; and *r*_*ALSPAC*_ = 0.33). A full overview of the performance of the Bohlin, EPIC overlap, and Knight gestational age clocks can be found in Supplementary Table S1 and **Supplementary Figures 1** and **2.**

### Associations between prenatal stress and epigenetic age estimates

Results from the meta-analysis showed that total prenatal stress was not significantly associated with epigenetic gestational age or measures of epigenetic age acceleration, as estimated by the Bohlin, EPIC overlap, or the Knight clock, all of which produced non-significant results. When testing for specific prenatal stress domains, namely life events, contextual risk, parental risk, and interpersonal risk, we again found no significant independent associations with any of the epigenetic age estimates or clocks. The results of the main model can be found in Table 2 and results of the extended models, which also indicated no significant associations, can be found in **Supplementary Table S1**. Cohort-specific results of just the Bohlin clock can be found in **Supplementary Table S3**. These are representative of the EPIC overlap clock and the Knight clock which also showed low correlations.

### Associations between prenatal stress and epigenetic gestational age estimates stratified by offspring sex

When stratifying analyses by sex, the same patterns were observed, with no associations of total prenatal stress, or any individual prenatal stress domain, with epigenetic clock estimates of gestational age in either boys or girls. The results of the main model can be found Table 3 in and results of the extended models can be found in **Supplementary Table S4**. Cohort-specific results of just the Bohlin clock stratified by sex can be found in **Supplementary Table S5**. These are again representative of the EPIC overlap clock and the Knight clock which also showed low correlations.

## Discussion

In this study, we pooled data from 3928 mother-offspring dyads from three prospective population-based cohorts and examined whether *in-utero* exposure to maternal psychosocial stress was associated with variation in offspring epigenetic gestational age at birth. We used comprehensive, harmonized measures of prenatal stress across cohorts, which enabled us to examine both the cumulative and independent effects of different stress domains. We also derived epigenetic gestational age estimates from three different clocks based on cord-blood DNAm profiles. The detailed information collected within the cohorts also allowed us to control for potential confounders including maternal pre-pregnancy BMI, prenatal smoking and alcohol consumption, highest education level attained and income, age at delivery, method of delivery and parity. The results of our meta-analysis indicate no statistically significant evidence of associations – a pattern that was consistent also within individual cohorts, across measures of prenatal stress, across different epigenetic clocks (Bohlin, Knight, EPIC-overlap), and when stratifying analyses based on offspring sex. Overall, our findings do not support a link between prenatal stress exposure and gestational epigenetic age (or age acceleration/deceleration) at birth in the general paediatric population.

These findings differ from previously published studies reporting a link between prenatal stress and either gestational epigenetic age deceleration ^26, 25, 19^ or acceleration [27] [21] [52]. Several factors may explain these discrepancies. First, unlike previous studies that have focused on single exposures (primarily maternal psychiatric symptoms), we used a broad measure of prenatal psychosocial stress, comprising a range of different exposures. Our rationale was that maternal psychiatric symptoms tend to co-occur with other stressors, which together cumulatively affect offspring health [53] and may partly exert their influence through shared biological pathways, including epigenetic programming. By using a cumulative score, however, any exposure-specific associations with gestational epigenetic clocks may have been obscured. To address this possibility, we also modelled different stress domains concurrently as predictors but did not observe any independent associations with epigenetic clock estimates. Although not statistically significant, it is worth noting that the direction of associations differed depending on the stress domain examined, with the parental risk domain (containing maternal psychopathology) showing consistent positive associations with epigenetic gestational age estimates across the different clocks, and the contextual risk domain (relating to socioeconomic stressors) showing consistent negative associations. Taken together, these results suggest that while pronounced stressor-specific effects are unlikely, subtle variations in associations with epigenetic gestational age estimates may exist and warrant further investigation.

A second reason for the observed discrepancies could be due to differences in the study samples. While we examined general population cohorts, where the prevalence of severe prenatal stress exposure is relatively low and most offspring are delivered at-term, previous studies have been primarily based on (single) selected, high-risk samples[54–58]. As such, we may not have been able to capture associations between prenatal stress and epigenetic clock estimates at birth, if these are evident only in premature children or at more severe ends of stress exposure. At the same time, our approach, involving - with stringent covariate adjustment, may have helped to reduce the likelihood of false positives. Furthermore, while our study included mother-child dyads of European descent, previous studies have been more varied, including samples from African, Hispanic, and South American populations. Current epigenetic gestational age clocks have been primarily developed using White individuals from Western Europe, and it is unclear whether they perform similarly across different ancestries. In the future, larger multi-cohort meta-analyses will be needed to detect subtle associations with greater power, enable the investigation of stressor-specific effects (while accounting for co-occurring stressors) and establish whether effects may vary according to sample characteristics, such as exposure severity and ancestry.

The statistically nonsignificant results reported herein do not detract from the important role of prenatal stress on development and health. Furthermore, the lack of associations with epigenetic gestational age clocks does not preclude the possibility that epigenetic patterns may still be involved as biological markers (and potentially mediators) of prenatal stress effects on foetal development. First, similar to first-generation epigenetic clocks used in adults, current gestational age clocks have been trained to predict chronological age and not biological *aging per se* – which may be more sensitive to prenatal stress. In the future, researchers may follow in the footsteps of second and third-generation adult clocks, which are trained to predict age-related phenotypes or the pace of aging (based on longitudinal aging markers), in order to build new gestational age clocks that are trained using early developmental phenotypes rather than chronological age. These could be tested whether these may associate more clearly to *in utero* environmental exposures. Second, prenatal stress may be associated with epigenetic changes at loci that are not included within gestational clocks. Indeed, several studies have reported associations between prenatal stressors and DNAm patterns in cord blood (for a review, see [59]). However, these findings also lack consistency; for example, a large meta-analysis of 12 independent studies of the Pregnancy And Childhood Epigenetics (PACE) consortium recently found no robust associations between prenatal maternal anxiety and DNAm in cord blood [60]. Finally, it is possible that, rather than relating to DNAm in cord blood, prenatal stress exposure associates with DNAm patterns in different tissues such as the brain, which is not accessible in vivo. Furthermore, epigenetic mechanisms other than DNAm, such as microRNAs or histone modifications (11,17)), which are also important – but currently under-researched – are also potential mediators of (prenatal) environmental effects on offspring health. ^51^

Our findings should be interpreted in the context of several limitations. First, as mentioned previously, the cohorts included in our study predominantly comprise White individuals from Western Europe and, as general population samples, they do not include high numbers of participants with psychiatric disorders or exposure to severe trauma/stress. Most participants also had a high socioeconomic status and education level. Therefore, these results could have limited generalizability to other populations, contexts, or more severe exposures – which is particularly relevant as many of the previous studies have focussed on high-risk groups of non-European descent. Second, while we carefully harmonized our cumulative measure of prenatal stress, the complexity and breadth of the measure (including a wide range of items clustered into distinct risk domains) meant that it was not identical between cohorts, showing slight variations in the tools used and the timing of measurements. Despite this, results were highly consistent across cohorts and there was little evidence of heterogeneity based on the results of the meta-analysis, suggesting that this is unlikely to explain null findings. Third, and relatedly, we relied on maternal self-reported measures of stressful exposures, as opposed to objectively assessed markers of physiological stress. Although our measure of prenatal stress has been previously shown to associate robustly with neurocognitive, psychiatric and physical health outcomes in offspring[53, 61] we cannot ascertain the extent to which this measure captures foetal exposure to stress. A third limitation of our study is related to the specific populations on which the epigenetic clocks were trained. For example, the Knight clock was developed using data from very premature infants, which may affect its accuracy and predictive capability in our general population samples, of primarily at-term children, as evidenced by the modest correlations with gestational age observed. However, no associations between prenatal stress and epigenetic age were identified across three different epigenetic gestational age clocks, suggesting that our results are not likely to be unduly influenced by the specific training features of each clock.

In summary, our findings indicate that maternal prenatal psychosocial stress exposure is not significantly associated with epigenetic gestational age or the extent to which it deviates from chronological gestational age. This suggests that the impact of prenatal maternal stress on developmental processes, as measured by current epigenetic gestational age clocks, might be less pronounced than previously thought, particularly within the general population where the prevalence of severe exposures is relatively low. Alternatively, the existing epigenetic gestational clocks may not be sensitive enough to capture subtle changes induced by *in utero* stress exposure. The study’s large-scale and comprehensive approach strengthens the reliability of these conclusions, advancing our understanding of the complex relationship between early-life stress and development.

## Figures and Tables

**Table 1.** Participant characteristics per cohort

Maternal characteristics	Generation R	MoBa 1	MoBa 2	ALSPAC

	N = 1,396^1^	N = 1212^1^	N = 678^1^	N = 642^1^

Age, years	32.2 (4.2)	30 (4.0)	30 (4.0)	29.9 (4.3)

Educational level				

No or primary	26 (1.9%)	40 (4.9%)	54 (8.1%)	<5 (<5%)^3^

Secondary	452 (33%)	207 (25%)	223 (33%)	542 (79%)

Higher	898 (65%)	576 (70%)	389 (58%)	146 (21%)

Parity				

Nulliparous	845 (61%)	567 (47%)	276 (41%)	301 (47%)

Multiparous	549 (39%)	645 (53%)	402 (59%)	336 (53%)

Pre-pregnancy body mass index	23.2 (3.8)	24.0 (4.3)	24.2 (4.5)	22.7 (3.4)

Mode of delivery				

Vaginal delivery spontaneous	1,025 (78%)	945 (78%)	521 (77%)	228 (57%)

Vaginal delivery induced	157 (12%)	133 (11%)	64 (9.5%)	5 (1%)

Caesarean section, elective	48 (3.7%)	43 (3.5%)	40 (5.9%)	<5 (<5%)^3^

Caesarean section, urgent	82 (6.2%)	91 (7.5%)	52 (7.7%)	<5 (<5%)^3^

Caesarean section, unspecified	0 (0%)	0 (0%)	0 (0%)	64 (16%)

Other	0 (0%)	0 (0%)	0 (0%)	101 (26%)^2^

Smoking				

Never smoked during pregnancy	969 (76%)	920 (76%)	500 (75%)	375 (63%)

Smoked until pregnancy was known	127 (9.9%)	182 (15%)	90 (13%)	159 (26%)

Continued smoking during pregnancy	184 (14%)	109 (9.0%)	80 (12%)	65 (11%)

Alcohol				

Never drank alcohol during pregnancy	370 (29%)	786 (66%)	345 (60%)	190 (30%)

Drank alcohol until pregnancy was known	196 (15%)	173 (15%)	77 (13%)	81 (13%)

Continued drinking alcohol during pregnancy	704 (55%)	227 (19%)	151 (26%)	359 (57%)

**Child characteristics**				

Sex				

Boy	708 (51%)	584 (48%)	385 (57%)	323 (50%)

Girl	688 (49%)	628 (52%)	293 (43%)	319 (50%)

Birth weight, gram	3,544 (510)	3,617 (551)	3,661 (535)	3,475 (491)

Clinically-estimated GA at birth, weeks	40.14 (1.5)	39.94 (1.9)	39.90 (1.6)	39.50 (1.6)

DNAm GA estimate, weeks				

Bohlin clock	39.29 (1.0)	41.02 (1..1)	40.42 (1.2)	39.50 (1.1)

EPIC overlap clock	39.47 (1.1)	40.14 (1.1)	39.71 (1.2)	39.71 (1.2)

Knight clock	36.32 (1.8)	41.23 (1.5)	39.37 (1.8)	38.30 (2.1)

Prenatal stress				

Total prenatal stress	0.3 (0.3)	0.7 (0.4)	0.8 (0.5)	<5 (<5%)^3^

Life events	0.1 (0.1)	0.1 (0.1)	0.1 (0.1)	<5 (<5%)^3^

Contextual risk	0.2 (0.2)	0.3 (0.2)	0.4 (0.3)	<5 (<5%)^3^

Parental risk	0.0 (0.1)	0.2 (0.2)	0.2 (0.2)	<5 (<5%)^3^

Interpersonal risk	0.1 (0.1)	0.1 (0.1)	0.1 (0.1)	<5 (<5%)^3^

1 Mean (SD); n (%); some data will not amount to total N due to missing values

2 In ALSPAC, delivery mode ‘other’ refers to forceps or vacuum extraction and assisted breech.

3 This may include zero.

**Table 2.** Meta-analysis results by epigenetic clock adjusted for clinically-assessed gestational age.

Epigenetic clock	Bohlin		EPIC overlap		Knight	

Prenatal stress	Beta (95% CI)	*P* value	Beta (95% CI)	*P* value	Beta (95% CI)	*P* value

Total prenatal stress	0.01 (−0.04, 0.06)	0.78	−0.01 (−0.07, 0.05)	0.75	−0.01 (−0.11, 0.09)	0.85

Life events	0.15 (−0.06, 0.35)	0.17	0.00 (−0.25, 0.24)	0.97	0.01 (−0.40, 0.42)	0.97

Contextual risk	−0.04 (−0.13, 0.04)	0.32	−0.05 (−0.15, 0.04)	0.26	−0.04 (−0.19, 0.11)	0.63

Parental risk	0.08 (−0.06, 0.22)	0.25	0.13 (−0.03, 0.28)	0.12	0.21 (−0.04, 0.46)	0.10

Interpersonal risk	−0.05 (−0.29, 0.18)	0.65	0.01 (−0.27, 0.29)	0.94	−0.28 (−0.75, 0.19)	0.24

Covariates included in the main model were child sex, cell-type proportions, and batch effects.

**Table 3.** Meta-analysis results of prenatal stress associations with epigenetic age estimate adjusted for clinically-estimated gestational age stratified by sex.

Epigenetic clock	Bohlin		EPIC overlap		Knight	

Prenatal stress	Beta (95% CI)	*P* value	Beta (95% CI)	*P* value	Beta (95% CI)	*P* value

Total prenatal stress						

Boys	0.02 (−0.05, 0.09)	0.64	−0.03 (−0.12, 0.05)	0.42	0.02 (−0.11, 0.15)	0.74

Girls	−0.01 (−0.09, 0.06)	0.78	0.01 (−0.07, 0.09)	0.83	−0.04 (−0.18, 0.10)	0.59

Life events						

Boys	0.06 (−0.24, 0.36)	0.70	−0.14 (−0.49, 0.21)	0.44	0.02 (−0.55, 0.60)	0.94

Girls	0.15 (−0.15, 0.45)	0.33	0.13 (−0.22, 0.47)	0.47	−0.07 (−0.66, 0.52)	0.81

Contextual risk						

Boys	−0.02 (−0.14, 0.10)	0.76	−0.05 (−0.15, 0.04)	0.26	0.05 (−0.17, 0.26)	0.68

Girls	−0.07 (−0.19, 0.05)	0.24	−0.08 (−0.22, 0.05)	0.23	−0.10 (−0.32, 0.12)	0.39

Parental risk						

Boys	0.10 (−0.11, 0.31)	0.34	0.11 (−0.12, 0.34)	0.34	0.22 (−0.14, 0.57)	0.24

Girls	0.08 (−0.12, 0.29)	0.43	0.14 (−0.08, 0.36)	0.21	0.17 (−0.20, 0.53)	0.37

Interpersonal risk						

Boys	−0.12 (−0.46, 0.22)	0.50	0.00 (−0.39, 0.39)	0.99	−0.22 (−0.88, 0.43)	0.51

Girls	−0.02 (−0.36, 0.33)	0.92	−0.09 (−0.51, 0.33)	0.67	−0.26 (−0.98, 0.46)	0.47

Covariates included in the main model were cell-type proportions and batch effects.
